# Exploiting the Innate Potential of Sorghum/Sorghum–Sudangrass Cover Crops to Improve Soil Microbial Profile That Can Lead to Suppression of Plant-Parasitic Nematodes

**DOI:** 10.3390/microorganisms9091831

**Published:** 2021-08-29

**Authors:** Roshan Paudel, Philip Waisen, Koon-Hui Wang

**Affiliations:** Department of Plant and Environmental Protection Sciences, University of Hawaii at Manoa, Honolulu, HI 96822, USA; rpaudel@hawaii.edu (R.P.); pwaisen@hawaii.edu (P.W.)

**Keywords:** allelopathic, biofumigation, microbial profile, no-till, root-knot nematode, soil health, *Meloidogyne incognita*, *Rotylenchulus reniformis*

## Abstract

Sorghum/sorghum–sudangrass hybrids (SSgH) have been used as a cover crop to improve soil health by adding soil organic matter, enhancing microbial activities, and suppressing soil-borne pathogens in various cropping systems. A series of SSgH were screened for (1) allelopathic suppression and (2) improvement of soil edaphic factors and soil microbial profile against plant-parasitic nematode (PPNs). The allelopathic potential of SSgH against PPNs is hypothesized to vary by variety and age. In two greenhouse bioassays, ‘NX-D-61′ sorghum and the ‘Latte’ SSgH amendment provided the most suppressive allelopathic effect against the female formation of *Meloidogyne incognita* on mustard green seedlings when using 1-, 2-, or 3-month-old SSgH tissue, though most varieties showed a decrease in allelopathic effect as SSgH mature. A field trial was conducted where seven SSgH varieties were grown for 2.5 months and terminated using a flail mower, and eggplant was planted in a no-till system. Multivariate analysis of measured parameters revealed that increase in soil moisture, microbial biomass, respiration rate, nematode enrichment index, and sorghum biomass were negatively related to the initial abundance of PPNs and the root-gall index at 5 months after planting eggplant in a no-till system. These results suggested that improvement of soil health by SSgH could lead to suppression of PPN infection.

## 1. Introduction

Root-knot nematodes (*Meloidogyne* spp.) are the most aggressive and damaging plant-parasitic nematodes (PPNs) on many crops [1]. The nematode-infected plants exhibit root galls where different life stages can be feeding inside. These sedentary endoparasites establish specialized feeding cells [2] that withdraw nutrients from the plants leading to a reduction in crop yield and quality [3]. Soil fumigation with synthetic chemicals is a popular and effective method of managing PPNs. However, the ban of potent fumigants such as methyl bromide and increasing restrictions on the use of other effective fumigant and non-fumigant nematicides have led to the search for more environmentally friendly and economically viable alternatives in recent years [4,5].

Brassicaceous cover crops such as brown mustard (*Brassica juncea*), rapeseed (*B*. *napus*), Ethiopian mustard (*B*. *carinata*), and white mustard (*Sinapis alba*) are known to produce biocidal isothiocyanates, volatile allelopathic compounds that can be used as biofumigants to suppress PPNs including root-knot nematodes (RKNs) [6], lesion nematodes (*Pratylenchus* spp) [7], and potato cyst nematodes (*Globodera* spp) [8,9]. Although the term biofumigation originally referred to brassica cover crops that release volatile compounds suppressive to soil-borne pests and pathogens [10], this practice has been extended to include non-brassica plants in recent years [11]. Sorghum (*Sorghum bicolor*) and its relatives contain dhurrin, a secondary metabolite in leaf tissues that can be converted to a highly toxic volatile compound known as hydrogen cyanide (HCN) or prussic acid [12]. Prussic acid poisoning in animals is related to the high affinity of cyanide ions to bind with the iron component of cytochrome oxidase molecule, thus preventing cellular respiration [13]. A similar mechanism of inhibition is also responsible for HCN’s nematicidal property [14,15].

Dhurrin (p-hydroxy-(S)-mandelonitrile-,8-D-glucoside) is a cyanogenic glucoside stored in the leaf epidermal cells of sorghum. It produces free HCN upon hydrolysis by an endogenous enzyme, *β*-glucosidase, in the mesophyll cells [16]. Sorghum and sudangrass (*S. sudanese*) have been used as biofumigant crops to control PPNs [11,17,18], particularly RKNs [19,20,21,22,23,24,25,26,27]. However, there are inconsistent abilities of sorghum varieties against root-knot nematode [28,29,30]. These could be due to the varieties tested, the environmental conditions, and cropping systems. Viaene and Abawi [31] discovered that the allelopathic effect of ‘Piper’ sudangrass against northern RKNs (*Meloidogyne hapla*) or their dhurrin content is higher at 1 and 2 months old compared to 3 months old, indicating that sorghum/sorghum–sudangrass hybrids (SSgH) have less allelopathic potential as the plant matures. However, terminating SSgH at 1 to 2 months old accumulates less carbon (C) biomass than at 3 months old. Identifying SSgH varieties that produce high dhurrin content independent of age would be beneficial.

Sorghum has been bred for photoperiod insensitivity to ensure a longer vegetative phase, which allows for more biomass production than conventional cultivars [32]. Energy sorghums are efficient at scavenging nitrogen in the soil and tolerant to environmental stress and accumulate a large amount of C, making them ideal for the biofuel industry [32,33]. Apart from industrial use, energy sorghum can also serve as a good soil builder, adding a large amount of organic matter into the soil [34]. Soil organic matter increases the biological activities in the soil and provides substrates and nutrients to soil microbes [35,36], which have a propensity to enhance nematode antagonistic microorganisms [37] or promote plant growth [38]. Adding soil organic matter can also alter soil edaphic properties such as soil carbon, cation exchange capacity, soil structure, and soil moisture [37]. Changes in soil edaphic factors by growing SSgH cover crops and how these changes shape the soil microbe–nematode relationship, which can lead to better suppression of PPNs, has hitherto not been studied and will be the foci of this research.

Soil health assessment is often performed by evaluating the physical, chemical, and biological properties of the soil. In the last few decades, nematodes have been used as bio-indicators of soil health [39,40]. The rationales for using nematodes as soil health indicators include the presence of nematodes in a different hierarchy of the soil food web; their ability to respond to changes in food resources; soil physical and chemical environment, soil disturbances such as tillage, fertilizer, or extreme climates; and the grouping of nematodes based on their trophic level and life-strategy [41]. Monitoring nematode abundance and community structure over time can help to provide insights into the ecological processes occurring in the soil and their impact on soil health [39,40,42,43]. Currently, the maturity index (MI), enrichment index (EI), channel index (CI), and structure index (SI) are used to describe soil food web-based on nematode faunal analysis. The channel index (CI) measures the weighted abundance of fungal feeders among the opportunistic nematode grazers on bacteria and fungi [44]. Thus, it represents the major decomposition pathway in the soil food web. The MI is calculated based on the colonizer–persister (cp) classification of the nematodes and provides an indication of the maturity of a soil food web [39], whereas, through calculation of weight abundance of nematodes with different cp values, EI assesses the soil food web response to resource enrichment, and SI shows the abundance of trophic linkages in the soil food web [45].

On the other hand, bacteria and fungi are the most abundant microorganisms in the soil, and they play a direct role in soil nutrient cycling, soil aggregation, antagonism to soil-born pests, and maintaining plant productivity. Therefore, besides assessing nematodes as soil health indicators, assessing soil microbial biomass could also provide additional information on the ecosystem functioning in the soil. Soil-dwelling bacteria are mostly involved in nitrogen cycling [46], whereas fungi are responsible for decomposing organic substrates with higher C: N ratios [47]. These microorganisms determine the process of organic matter synthesis and turnover [48]. In addition, plant-growth-promoting rhizobacteria (PGPR) and mycorrhizal fungi are widely studied for their ability to suppress soil-borne pathogens and improve plant health [49].

Microbial abundance or biomass in the soil can be estimated using culture-independent methods such as phospholipid fatty acid (PLFA) analysis and DNA-based approaches [50]. Using PLFA as biomarkers for microbial identification [48] provides information on the soil microbial community biomass and a quantitative indicator of soil health. Gram-positive bacteria are represented by saturated PLFAs, whereas Gram-negative bacteria are represented by monounsaturated PLFAs. Actinomycetes are characterized by mid-chain branching saturated PLFAs. The estimation of arbuscular mycorrhizae involves the use of a monounsaturated fatty acid biomarker [51]. PLFA analysis has been used to study the soil microbial community affected by different agricultural management practices [52]. Although metagenomic analysis resolves soil microbiomes to a finer taxonomic level, it often could not provide a full understanding of the ecosystem functioning of each taxon. Geisen et al. [53] recommended PLFA analysis for distinct ecosystem functioning in the soil and DNA amplicon sequencing for ecosystem diversity analysis. The current research further explores the relationships between nematode community indices with microbial profiling and potential association between PPN suppression with soil physical and chemical properties.

It was hypothesized that (1) the allelopathic potential of SSgH is affected by the age and variety of SSgH; (2) cover cropping with SSgH can improve soil edaphic properties and nematode soil health indicators; and (3) more abundant microbial activities and a more diverse microbial profile would lead to better suppression of PPNs. Specific objectives of this research were to (i) understand the effect of SSgH variety and plant age on the allelopathic effect of SSgH against *M. incognita*, (ii) examine the effects of no-till SSgH cover cropping on the population dynamic of soil microbial profiles and nematode soil health indicators, and (iii) investigate the relationship between soil health indicators and PPN suppression.

## 2. Materials and Methods

### 2.1. Effect of SSgH Variety and Age on the Allelopathic Effect against M. incognita

**Figure 1:**
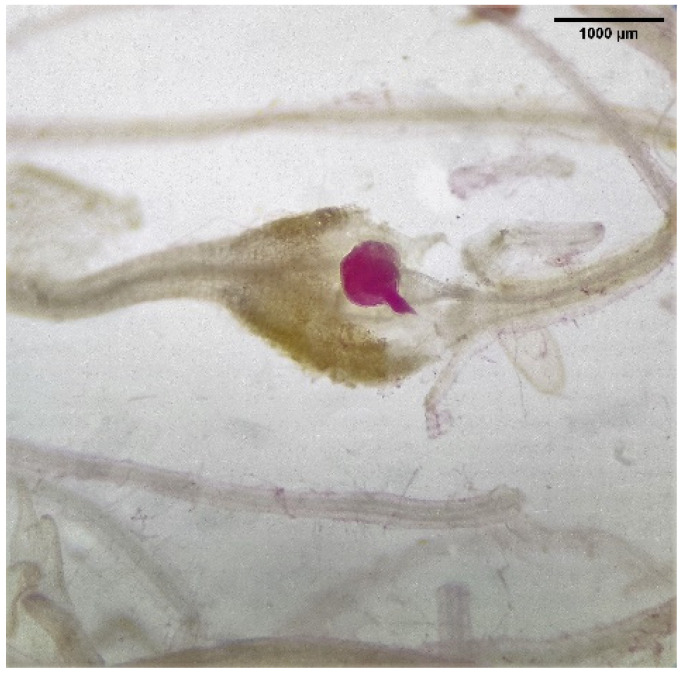
Roots were stained at 1 month after inoculation to quantify infection rates of female RKNs.

Two greenhouse pot experiments were conducted to screen SSgH varieties that are most suppressive against *M. incognita*. The first greenhouse trial (Trial I) was conducted on 27 March 2020 where residues of 11 SSgH varieties: ‘Elite Brown Mid Rib’, ‘Bundle King’, ‘Monster II’, ‘Big Kahuna Plus’, ‘Cow Vittles II’, ‘512 × 14’, ‘Latte BMR’, ‘535 × 14’, ‘Latte’, ‘NX 4264’, and ‘NX-D-61’ along with ‘Tropic Sun’ sunn hemp (*Crotalaria juncea*) were amended into the soil. A no-amendment treatment was included as a negative control. The second trial (Trial II) was conducted on 8 June 2020, repeating Trial I with additional sudangrass varieties, ‘Piper’ and forage sorghum ‘EBMR’. All test plants were grown in the field at Magoon Teaching Facility, University of Hawaii at Manoa. Shoot biomass of all test plants was collected at 1, 2, and 3 months after planting and brought in to set up the greenhouse trials. Fresh shoot tissues were chopped into small pieces of 1 cm consistency prior to amending into the soil at 1% (*w/w*) dry weight equivalent. Each 80 cm^3^ Ray Leach Cone-tainer (Stuewe and Sons, Inc., Tangent, Oregon) consisted of 103 g dry weight of sterile sand: soil mix (1:1 *v/v*). For both trials, sterile soil was an autoclaved Wahiawa soil (Tropeptic Eutrustox, clayey, kaolinitic, isohyperthermic soil) collected from Poamoho Experiment Station. Shoot tissues and sterile soil were placed in a plastic bag and thoroughly mixed before transferring into each pot. Experiment was arranged in a 12 × 3 (amendment × plant age) factorial design with four replications on a greenhouse bench. Five-week-old ‘Hirayama’ kai choi (*Brassica juncea*) seedlings were transplanted into each pot, representing a replication, and 220 J2 of *M. incognita*/plant was inoculated on the same day. The 1-, 2- and 3-month age experiments were conducted from 29 May 2020, 14 July 2020, and 12 August 2020, respectively, for Trial I and from 8 August 2020, 20 September 2020, and 14 November 2020, respectively, for Trial II. Average day/night hours were 13:10/10:50 and 11:58/12:02 for Trial I and Trial II, respectively. A WatchDog temperature data logger was buried at 5 cm deep in the soil inside a Cone-tainer during both trials. The average, maximum, and minimum temperatures for Trial I were 26.3, 31.2, and 21.4 °C; whereas those in Trial II were 27.5, 33.0, and 22.7 °C. Plants were watered daily using a sprinkler irrigation system. Pure culture of *M. incognita* was obtained from ‘Orange Pixie’ tomato (*Solanum lycospersicum*) plants grown at Magoon Greenhouse, University of Hawaii at Manoa. Nematode eggs were extracted from tomato roots using a 0.6% sodium hypochlorite solution [54] followed by a centrifugal sugar flotation method [55]. Eggs were hatched in Baermann trays at 24 °C for 14 days before use [56]. Root penetration by the nematodes was observed 1 month after soil amendment by staining a 0.3 g random subsample of kai choi roots per pot using acid fuchsin [57]. Stained roots were observed under a dissecting microscope (Leica Microsystems Company, Wetzlar, Germany) to quantify the numbers of J2, J3–4, and females per sample (Figure 1).

### 2.2. Effect of SSgH Varieties on Soil Edaphic Factors, Nematode Community, and Microbial Profile

A field trial was conducted on 28 May 2020 at Poamoho Experiment Station, Waialua, HI (21°33′08.3″ N 158°06′08.4″ W) to compare SSgH varieties for their potential to improve soil health. The soil at the test site was a Wahiawa Soil Series described in Section 2.1 with 18.6% sand, 37.7% silt, 43.7% clay, and pH 6.7. The field site was naturally infested with *M. incognita, M. javanica,* and *Rotylenchulus reniformis* (Table S1). Seven SSgH varieties with an assortment of allelopathic effects against RKN penetration and development based on the greenhouse experiment results were tested (Table 1). A bare ground (BG) was included as an untreated control. Each SSgH variety was seeded at 56 kg seeds/ha in 3.6 × 1.2 m^2^ plots. Experimental plots were arranged in a randomized complete block design (RCBD) with four replications. Based on the results from the greenhouse studies, cover crops were grown for 2.5 months and terminated in a no-till system using a flail mower operated by a BCS walk-behind tractor (Model 853, BCS America, LLC, Portland, OR, USA). The mower had a plastic flap to contain the flailed tissues within each plot. Prior to the termination of SSgH, the biomass from each plot was estimated using three 0.1 m^2^ quadrants. Each plot was 0.5 m away from the adjacent rows, with a 1.5-m bare area between plots in a row. A total of 32 plots were established. Six-week-old ‘Shikou’ eggplant (*Solanum melongena*) seedlings were transplanted on 10 August 2020 with minimal disturbance to the soil. Each plot had seven eggplant seedlings planted in alternate at 0.5 m spacing between plants in a plot. Eggplants were fertilized using 73 kg N/ha of Suståne 8-2-4 organic fertilizer (Suståne Natural Fertilizer, Inc., Cannon Falls, MN, USA) and drip-irrigated. The experiment was terminated at 4.5 months after eggplant transplanting as the plants began to senesce.

#### 2.2.1. Nematode Community Analysis

From the field trial described in Section 2.2., approximately 1000 cm^3^ soil samples were systematically collected from four cores per plot in a zigzag pattern from the top 10 cm of soil using a GroundShark shovel (Forestry Suppliers Inc., Jackson, MS, USA) at the initiation of the experiment and at monthly intervals thereafter from cover crop planting and throughout eggplant growth. All soil samples collected were sieved through a 0.5 cm^2^ mesh screen and homogenized, and one 250 cm^3^ soil subsample per plot was extracted for nematodes using elutriation and the centrifugal floatation method [55,58]. All nematodes extracted were identified to the genus level wherever possible and counted under an inverted microscope (Leica DMIL, Leica Microsystems Company, Wetzlar, Germany). Nematodes were assigned to respective trophic groups (algivores, bacterivores, fungivores, herbivores, omnivores, or predators) according to Yeates et al. [59]. Nematode richness was calculated as the total number of different taxa recorded per sample. The Simpson index of diversity [60]; the maturity index (MI) [39]; and EI, SI, and CI [45] were then calculated.

#### 2.2.2. Soil Quality Analysis

Soil subsamples collected 2 weeks after cover crop termination and 3 months after planting eggplant were submitted to the Agricultural Diagnostic Services Center (ADSC) of the University of Hawaii, Honolulu, Hawaii to analyze for total C and N content using LECO TruSpec CN (LECO Corporation, Saint Joseph, MI, USA).

FieldScout TDR 100 Soil Moisture Meter (Spectrum Technologies INC., Aurora, IL, USA) was used to measure volumetric soil moistures twice during the eggplant growing season with 12 cm rods buried 10 cm deep in the rhizosphere from three randomly selected spots per plot. Soil from each plot was measured for infiltration rate at 2 months after growing sorghum and 3 months after planting eggplant using a double-ring infiltration method [61].

#### 2.2.3. Soil Microbial Profiling

A soil sample was collected from the rhizosphere of SSgH or eggplants from three plants/plot at 2 weeks after SSgH termination and 3 months after planting eggplant for PLFA analysis. To obtain rhizosphere soil, roots were dug out and shaken in a bucket to remove major soil clumps and collect rhizosphere soil by screening through a 0.5 cm^2^ mesh metal sieve. A 10 g subsample from the composited rhizosphere soil of each plot was placed in a 14 mL Falcon tube (Becton Dickinson, Lakes, NJ, USA) and immediately stored in a cooler packed with dry ice. Soil samples were transported to the laboratory and stored at −80 °C (PHCBI, cat. No. MDF-DU702VHA-PA, PHC corporation, Wood Dale, IL, USA) before shipping to Microbial ID Laboratory (MIDI Inc., Newark, DE, USA) for PLFA analysis.

#### 2.2.4. Eggplant Plant Response

Following the termination of the SSgH, eggplant plant height was monitored at 5 weeks after transplanting eggplant. Eggplant fruits were harvested from four plants per plot beginning at 2 months after planting, and weekly thereafter. Total fruit numbers and fruit weight per plot were recorded. Fruits were sorted into marketable and unmarketable categories. Unmarketable fruits were due to thrips and mites damage. At the end of the experiment, around 4.5 months after transplanting, three plants from each plot were uprooted, and roots were washed, weighted, and rated for root-gall index (RGI) based on a 0–10 scale by Netscher and Sikora [62].

#### 2.2.5. Statistical Analysis

All data were checked for normality using PROC UNIVARIATE in SAS 9.4 (SAS Institute Inc., Cary, NC, USA). Data were log(x + 1) or square root transformed when needed. Data from the greenhouse trials were subjected to a 12 × 3 factorial analysis of variance (ANOVA) in Trial I and 14 × 3 ANOVA for Trial II. For the field trial, soil quality parameters and nematode community analysis data after initiation of the experiment were subjected to repeated measure and one-way analysis of variance (ANOVA) using PROC GLM in SAS 9.4. If no interaction between treatment and sampling date occurred, the means of treatment were separated by Waller–Duncan *k*-ratio (*k* = 100) *t*-test wherever appropriate. Since PLFA data were only collected twice, once at 2 weeks after cover crop termination and another at 3 months after eggplant planting, these data were subjected to one-way ANOVA by date. All parameters collected were subjected to canonical correspondence analysis (CCA) twice using CANOCO for Windows 4.5 [63]. The first CCA was from data collected from the initiation of the experiment (28 May 2020) to 2 weeks after cover crop termination (27 August 2020), whereas data from 28 May 2020 to 20 January 2021 (3 months after eggplant planting) were subjected to the second CCA.

## 3. Results

### 3.1. Effect of SSgH Variety and Plant Age on M. incognita Suppression

**Figure 2:**
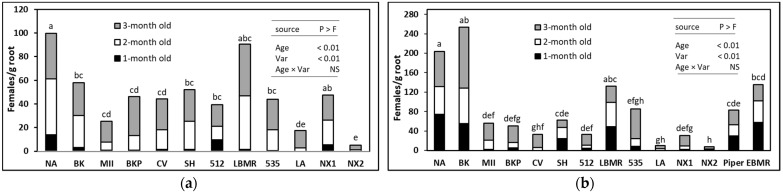
Effect of 1-, 2-, and 3-month-old SSgH biomass on the number of root-knot nematode females on mustard green roots in Trial I (**a**) and Trial II (**b**). NA (no amendment), BK (Bundle King), MII (Monster II), BKP (Big Kahuna Plus), CV (Cow Vittles), SH (sunn hemp), 512 (512 × 14), LBMR (Latte BMR), 535 (535 × 14), LA (Latte), NX1 (NX 4264), and NX2 (NX-D-61). Means (*n =* 12) followed by the same letter(s) are not different according to Waller–Duncan *k*-ratio (*k* = 100) *t*-test.

In both greenhouse experiments terminated at 1 month after *M. incognita* inoculation, no significant differences (Trial 1: *F* = 1.22, df = 11, *p* > 0.05; Trial 2: *F* = 1.53, df = 13, *p* > 0.05,) in the number of J2 or J3–J4 juveniles per g roots were detected by varieties, but significant differences were detected among varieties in numbers of females developed. Thus, only the numbers of females/g root were presented. Two-way ANOVA revealed significant differences in females/g root by plant age, but no significant interaction (*p >* 0.05) between plant age and SSgH variety was detected in both trials. In Trial I, energy sorghum ‘NX2’ was most suppressive (*F* = 6.19, df = 11, *p* *≤* 0.05) to *M. incognita* female development for all three ages of sorghum biomass amended compared to the no-amendment control (Figure 2a). ‘LA’ was suppressive to *M. incognita* when using 1- and 2-month-old tissue; however, it could not suppress the number of females when using 3-month-old biomass (Figure 2a). In Trial II, ‘NX2’ and ‘LA’ were again the most suppressive to *M. incognita* female development compared to the control (Figure 2b). Interestingly, ‘NX2’ and ‘LA’ suppressed the number of females more effectively than the sunn hemp amendment (Figure 2a,b). Results from both trials showed a clear trend of a decrease in the allelopathic effect of SSgH against *M. incognita* as the age of the biomass increased (Table 2).

### 3.2. Effect of No-Till SSgH Cover Cropping on Soil Edaphic Properties

**Figure 3:**
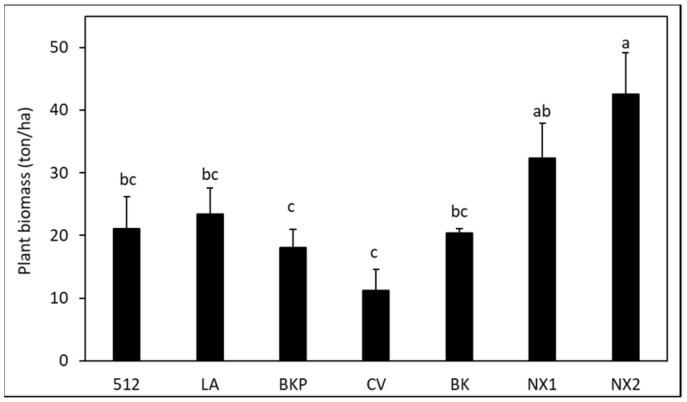
Biomass of SSgH generated from 512 (512 × 14), LA (Latte), BKP (Big Kahuna Plus BMR), CV (Cow Vittles), BK (Bundle King), NX1 (NX 4264), and NX2 (NX-D-61) in the field. Means (*n =* 4) followed by same letter(s) are not significantly different according to the Waller–Duncan *k*-ratio (*k* = 100) *t*-test.

At the termination of the cover crop in the no-till field trial, significant differences in cover crop biomass were detected among the varieties (*F* = 6.33, df = 6, *p ≤* 0.05). Energy sorghum ‘NX2’ produced higher biomass *(p* *≤* 0.05) than other SSgH varieties (Figure 3). In general, energy sorghum (NX1 and NX2) produced more biomass than forage sorghum (BK, BKP, and CV), with biomass of sorghum–sudangrass hybrids (512 and LA) weighted in between.

**Figure 4:**
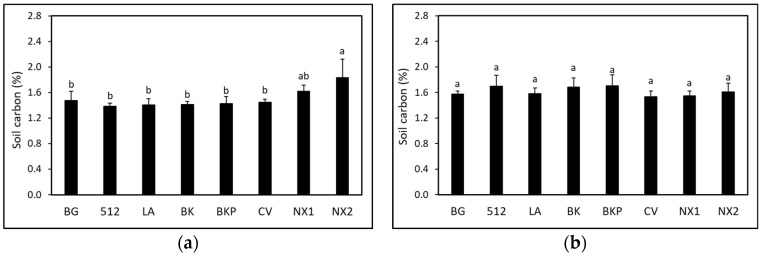
Effect of SSgH on soil carbon measured at (**a**) 2.5 months after planting cover crop and (**b**) 3 months after eggplant planting. Values with the same letter(s) are not significantly different according to Waller–Duncan *k*-ratio (*k* = 100) *t*-test. BG (BG control), 512 (512 × 14), LA (Latte), BK (Bundle King), BKP (Big Kahuna Plus BMR), CV (Cow Vittles), NX1 (NX 4264), and NX2 (NX-D-61).

**Figure 5:**
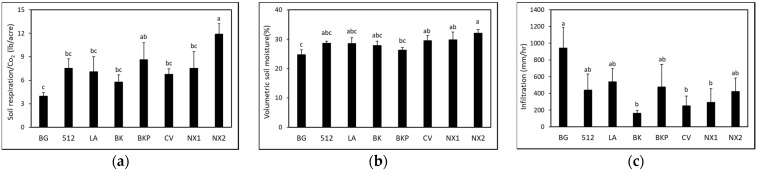
Effect of SSgH on (**a**) soil respiration, (**b**) volumetric soil moisture, and (**c**) infiltration rate. Means (*n =* 4) followed by the same letter(s) are not different according to Waller–Duncan *k*-ratio (*k* = 100) *t*-test.

Soil carbon was significantly higher (*F* = 2.39, df = 7, *p* *≤* 0.05) in ‘NX2’ sorghum compared to the BG control at 2.5 months after planting SSgH cover crop (Figure 4a). All other SSgH treatments did not increase soil carbon at the time of termination of the cover crop. At 3 months after eggplant planting, soil carbon was not different (*p >* 0.05) among SSgH varieties, and all SSgH treatments were not different from the BG control (Figure 4b). No significant interaction was found between treatments and dates for soil respiration, volumetric soil moisture, or infiltration rate. Thus, data were pooled across dates. Energy sorghum ‘NX2’ and forage sorghum ‘BKP’ had higher (*F* = 3.25, df = 7, *p* *≤* 0.05) soil respiration rates compared to the BG control (Figure 5a). Soil moisture was higher in ‘NX2’, ‘NX1’, and ‘CV’ sorghum compared to the BG control (Figure 5b). However, the water infiltration rate, though highly variable, was not different between treatments (Figure 5c).

### 3.3. Effect of SSgH on Soil Nematode Communities

**Figure 6:**
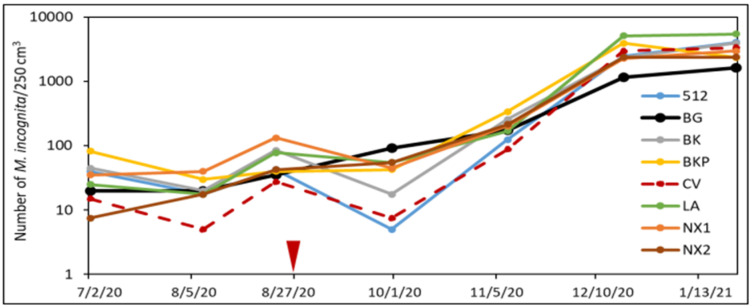
Number of *Meloidogyne* spp. (*n* = 4) juveniles in the soil at each sampling date from sorghum/sorghum–sudangrass cover crop plots throughout cover cropping and eggplant growing period in a no-till field trial. 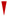 indicates two weeks after cover crop termination and eggplant planting time.

No significant interaction was found between treatments and sampling date; thus, all nematode data throughout the SSgH–eggplant cropping cycle were pooled and subjected to repeated measure analysis. Whereas SSgH treatment did not affect the abundance of reniform nematodes (*Rotylenchulus reniformis*), the population density of RKNs (*Meloidogyne* spp.) was numerically lowest in the BG, but statistically lower in ‘CV’ than BG (Table 3). However, the abundance of root-knot nematodes was only higher in most SSgH than BG toward the end of the eggplant growing cycle (Figure 6). The number of RKNs in the soil remained low in ‘CV’ until 3 months after eggplant planting. The abundance of bacterivorous nematodes was increased by ‘512’ (*F* = 2.05, df = 7, *p* *≤* 0.05), whereas omnivorous nematodes were increased by ‘NX2’, ‘BKP’, ‘BK’, and ‘512’ (*F* = 2.38, df = 7, *p* *≤* 0.05) compared to BG (Table 3). In general, all SSgH treatments numerically increased the abundance of omnivorous nematodes compared to BG. In terms of nematode community indices, SSgH treatments only affected richness and CI. All SSgH treatments increased nematode richness compared to BG (*F* = 3.95, df = 7, *p* *≤* 0.05) except for ‘CV’ and ‘BKP’. There was also a trend that all SSgH increased CI compared to BG, but this was most significant in ‘BK’, ‘BKP’, and ‘NX2’ (*F* = 1.71, df = 7, *p* *≤* 0.05). Though not significant, all SSgH also increased SI compared to the BG (Table 3).

### 3.4. Effect of SSgH on Soil Microbial Profile

At 2 weeks after SSgH termination, microbial biomass represented by total phospholipid fatty acids (TPLFA) was increased (*F* = 5.53, df = 7, *p* *≤* 0.05) by six SSgH treatments excluding ‘NX1’ sorghum (Table 4). ‘LA’ and ‘NX2’ showed the most promising results with 92% and 60% higher microbial biomass, respectively, than BG. Energy sorghum ‘NX2’ significantly increased microbial biomass of non-arbuscular mycorrhizal fungi and % eukaryotes compared to BG (Table 4). BG had higher GP/GN, S/U, and M/P ratios, whereas, ‘NX2’ had the highest F/B and PD/PR.

Three months after eggplant planting, ‘LA’ still increased (*F* = 2.38, df = 7, *p ≤* 0.05) total microbial biomass by 87% more than BG (Table 4). Although not statistically significant, ‘CV’ and ‘NX2’ showed 45% and 38% increases in microbial biomass, respectively, compared to BG. Relative abundance of Gram-positive bacteria was highest (*p ≤* 0.05) in BG. Abundance of eukaryotes was increased (*F* = 9.81, df = 7, *p ≤* 0.05) by ‘LA’, ‘BK’, and ‘BKP’. In terms of microbial ratios, all SSgH had lower GP/GN than ‘BG’ (*F* = 7.01, df = 7, *p ≤* 0.05) except for ‘NX1’. ‘NX1’ also had higher M/P compared to BG. A higher (*F* = 5.35, df = 7, *p ≤* 0.05) PD/PR ratio was found in ‘BK’, ‘BKP’, and ‘LA’.

### 3.5. Relationships between Soil Health Indicators and Plant-Parasitic Nematode

**Figure 7:**
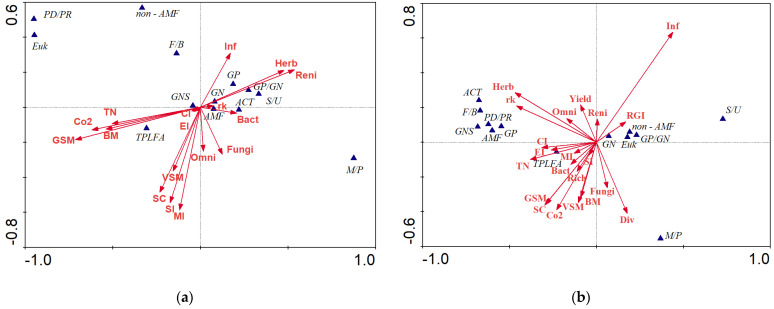
Canonical correspondence analysis (CCA) biplots showing the relationships among species variables (blue triangles) and environmental variables (red arrows) at (**a**) the time of cover crop termination and (**b**) at 3 months after eggplant planting. The first two axes explained 87.4% and 86% of the variation in the data in (**a**,**b**), respectively. The species variables include total phospholipid fatty acid (TPLFA), arbuscular mycorrhizal fungi (AMF), Gram-negative bacteria (GN), Gram-positive bacteria (GP), actinomyces (ACT), eukaryotes (EUK), non-arbuscular mycorrhizal fungi (non-AMF), fungi/bacteria (F/B), predator/prey (PD/PR), Gram-positive bacteria/Gram-negative bacteria (GP/GN), saturated fatty acids/unsaturated fatty acids (S/U), monounsaturated fatty acids/polyunsaturated fatty acids (M/P), Gram-negative stress (GNS). Environmental variables include reniform nematode (Reni), root-knot nematode (rk), herbivores/PPNs (Herb), bacterivores (Bact), fungivores (Fungi), omnivores (Omni), nematode richness (Rich), nematode diversity (div), maturity index (MI), enrichment index (EI), structure index (SI), channel index (CI), soil infiltration rate (Inf), soil carbon (SC), eggplant yield (Yield), soil respiration (Co2), volumetric soil moisture (VSM), gravimetric soil moisture (GSM), SSgH tissue nitrogen (TN), SSgH biomass (BM), and root-gall index (RGI).

Canonical correspondence analysis (CCA) was performed between 13 species variables and 17 environmental variables collected at the time of cover crop termination (Figure 7a) and 13 species variables and 21 environmental variables recorded at 3 months after eggplant planting (Figure 7b). The first two canonical axes explained 87.4% and 86% of the variation for the two ordination plots, respectively (Figure 7a,b). At 2 weeks after SSgH termination, most of the soil health indicators including total microbial biomass (TPLFA), soil microbial respiration rates, soil moisture (GSM, VSM), soil carbon (SC), nematode enrichment index (EI), maturity index (MI), structure index (SI), and abundance of omnivorous nematodes were negatively related to the abundance of PPNs and reniform nematodes (Figure 7a). Water infiltration rate (Inf) was negatively correlated to the above-mentioned soil health indicators but positively correlated to the abundance of Gram-positive bacteria (GP), Gram-negative bacteria (GN), and plant-parasitic nematode (Herb). At 3 months after eggplant planting, a negative relationship between root-gall index (RGI) on eggplant with TPLFA, soil moisture, soil carbon, sorghum biomass (BM), soil respiration, SSgH tissue nitrogen content (TN), nematode richness (CI, EI, MI, and SI), and abundance of bacterial feeding nematodes was observed (Figure 7b). Eggplant yield and infiltration rate, on the other hand, were negatively correlated to many of the above-mentioned parameters. The abundance of PPNs was positively correlated with the abundance of actinomycetes (ACT), Gram-negative stress (GNS) bacteria (i.e., a ratio of cyclopropyl fatty acids, a type of saturated fatty acids, to unsaturated fatty acids present in G-ve bacteria), arbuscular mycorrhizal fungi (AMF), Gram-positive (GP) bacteria, and ratios of the abundance of predators to prey (PD/PR) and fungi to bacteria (F/B) (Figure 7b).

### 3.6. Eggplant Growth and Yield

Eggplant height recorded at 5 weeks after transplanting was not significantly different (*p >* 0.05, df = 7, *F* = 0.69) among treatments (Table 5). Total eggplant fruit weight was numerically higher in all SSgH treatments compared to BG control, though not significant (*p >* 0.05). A higher fruit number was observed in ‘CV’ plots. Root weight was increased (*F* = 1.86, df = 7, *p* *≤* 0.05) by ‘BK’ sorghum, and all SSgH treatments had higher root weight than BG. On the other hand, there were no differences (*p >* 0.05) among treatments for root-gall formation.

## 4. Discussion

### 4.1. Allelopathic Potential of SSgH

The decline in allelopathic effects of SSgH against *M. incognita* observed in this study is consistent with previous studies that reported a decline in dhurrin concentration in sorghum as the plants mature [20,64]. The reduction in allelopathic effect of SSgH in older plants would suggest the need for an early termination of SSgH cover crop to ensure effective suppression of RKNs, which could reduce the efficacy of SSgH for building soil organic matter and improving other aspects of soil health. However, since 3-month-old plant tissue of ‘NX2’ and ‘LA’ were equally effective in suppressing root-knot nematode compared to younger plant tissues, this would warrant growing these cover crops for 3 months to accumulate higher cover crop biomass. Among all the SSgH tested, ‘NX2’ and ‘LA’ had the highest suppression against the development of *M. incognita* females; thus, these varieties were reevaluated in the subsequent field trial. Based on the allelopathic results from the greenhouse pot trials and the observation of biomass production of SSgH over time where the biomass increased by up to sevenfold when grown for 2 months compared to 1 month and up to twofold from 2 to 3 months, we determined that we should terminate the SSgH cover crop in the field trial for more than 2 months to ensure biomass production but less than 3 months to maximize the allelopathic effects of certain varieties in the field trials.

Unfortunately, the allelopathic effect of SSgH including that from NX2 and LA against RKNs was not observed in the no-till field trial. This could be because biofumigants in the no-till condition did not get in contact with soil nematodes for long enough to provide the same level of control as in the greenhouse pot trials where SSgH tissues were amended in the soil. These observations are in line with previous studies using brassicaceous cover crops for biofumigation where tissue maceration plus soil incorporation treatment was the most effective in suppressing PPNs compared to no-till treatment [11,65]. The biofumigant produced by SSgH is HCN, which is highly volatile [66]. Therefore, it is important to incorporate the biofumigant into the soil to come in contact with PPNs, as well as prolonging the allelopathic activity of HCN. Unlike the root-knot nematode population, which showed a continuous increase toward the end of eggplant season, the reniform nematode population did not show a clear trend. Future research should investigate terminating the SSgH cover crop by strip-till. Nonetheless, the no-till field trial showed that none of the SSgH tested are hosts to *Meloidogyne* spp., as the number of RKNs in the soil was lower than 100 per 250 cm^3^ and was not different from initial sampling. This is consistent with Djian-Caporalino et al. [20] finding that the ‘Piper’ (low dhurrin) and sudangrass hybrid ’270911’ (high dhurrin) was a poor host of *M*. *incognita*.

### 4.2. Effects of SSgH Cover Crop on Soil Edaphic Properties and Soil Health Indicators

As expected, SSgH cover cropping enhanced various soil edaphic properties, microbial biomass (TPLFA, non-AMF and % eukaryotes), and nematode soil health indicators (abundance of omnivorous and bacterivorous nematodes, richness, and CI) in the no-till field trial. Based on CCA, higher cover crop biomass in this trial led to higher soil carbon, which would lead to a higher macroaggregate stability, which creates a suitable habitat for various soil microorganisms [67]. Data here also showed that soil organic matter was positively related to volumetric soil moisture and total soil microbial biomass (TPLFA). Soil organic matter, especially in a no-till system, provides a stable food supply to the microorganisms [68,69,70]. The higher total microbial biomass in SSgH compared to the BG was observed at 2 weeks as well as at 3 months after SSgH cover crop termination.

However, a shift in microbial population over time was observed. Initially, non-AMF, % eukaryotes, and F/B responded positively to SSgH, whereas GP/GN, S/U, and M/P were reduced by SSgH cover cropping. This is no longer obvious at 3 months after SSgH termination. SSgH could have released carbon and secondary metabolites via their root exudates that could support more fungal microbial activity [67]. Soon after cover crop termination, non-AMF was particularly higher in CV and NX2 than BG. These saprophytic fungi play an important role in decomposing high C: N ratio organic residues in the soil and help in improving soil structure through the secretion of extracellular enzymes and by physically binding soil particles together with their hyphal mass, rendering the soil more stable [70]. Hence, the overall F/B ratio was higher in the no-till SSgH plots compared to the BG, particularly for CV and NX2 soon after cover crop termination. Organically managed and undisturbed ecosystems tend to have a higher F/B of microbes than conventionally managed disturbed soil ecosystems [71,72]. This study also concurs with that by Blaise et al. [73], where they found sorghum, sunn hemp, or desmodium (*Desmodium triflorum*) intercropped with wide-row spacing *Bt* corn, as living mulch supported higher soil biological activities such as basal respiration, microbial biomass carbon, and soil enzymes than the other mulch treatments. Similarly, the current study also showed that soil respiration was higher in SSgH (particularly NX2 and BKP) than BG throughout the eggplant cropping cycle.

While fungi are associated with macroaggregates, the majority of the bacteria live within microaggregates [74,75]. However, bacteria were separated into two major functional groups, Gram-positive (GP) and Gram-negative (GN) bacteria. A decrease in GP/GN by all SSgH except NX1 compared to BG in both PLFA analysis 2 weeks or 3 months after cover crop termination is encouraging as this indicates a lower-stress soil microbial community. This is because GP bacteria are oligotrophic, utilizing recalcitrant carbon sources, and GN bacteria tend to be copiotrophs surviving on labile carbon [76,77]. SSgH must have been conditioning the soil with more labile C, thus increasing GN bacteria and reducing GP/GN. A value of GP/GN closer to 1 indicates a balanced bacterial community and greater diversity. At both PLFA sampling, BG had GP/GN further from 1 than most SSgH, indicating less-balanced and lower diversity of bacterial communities.

The abundance of protists (depicted as % eukaryotes in CCA), a diverse group of unicellular eukaryotes excluding plant, animal, and fungus, was increased by most SSgH cover crops except for BK at 2 weeks after cover crop termination. These protists, including amoebas, ciliates, diatoms, plasmodium, oomycetes, and slime molds, could provide rapid nutrient turnover through the consumption of smaller microorganisms such as bacteria and fungi, change in microbial species composition through selective feeding, and the production of certain antibiotics that may protect plants against diseases [78,79]. Thus, an increase in % eukaryotes is a good property to possess following SSgH termination. While the increase in eukaryotes varied at 3 months after eggplant planting, a higher PD/PR (i.e., predator/prey or protozoa/bacteria) observed in most SSgH treatments except NX1 suggested a continuation in more nutrient turnover (mineralization) by protists even at 3 months after SSgH cover crop termination. Haubert et al. [80] also suggested that organic cover cropping sustained a diverse group of organisms including those in higher trophic levels than conventional farming without much organic input.

PLFA can be calculated into a ratio of saturated to unsaturated fatty acids (S/U), where higher ratios correspond to increasing stress [81,82]. Lower S/U in most SSgH treatments except NX1 compared to BG at 2 weeks and 3 months after cover crop termination corresponded to the increase in C and water availability by SSgH treatments compared to BG.

### 4.3. Relationship between Nematode Indicators with Soil Edaphic Factors, PPNs, and Crop Yield

While microbial biomass profile responded to SSgH treatment in the no-till system rather well within one cropping cycle of SSgH-eggplant planting, responses from the nematode community analysis were sparse. Only the abundance of bacterivores and omnivores and richness and CI differed between BG and some of the SSgH treatments. This is partially expected since nematodes are higher up in the soil food web from soil microbes and would take longer to respond to no-till organic management. Hinds et al. [83] noticed a difference in nematode community changes using sunn hemp as a cover crop where the response was faster in the strip-till system vs. the no-till system. Wang et al. [84] also reported a change in nematode community structure within one cropping cycle after sunn hemp strip-till cover cropping. Despite the weak response of nematode community indices to SSgH no-till treatments, the abundance of omnivorous nematodes, EI, CI, MI, and SI, and richness (rich) and diversity (Div) were positively related to changes in soil edaphic factors (GSM, VSM, SC, and BM) throughout the eggplant cropping cycle. However, their negative relationship with the water infiltration rate (Inf) deviated from the assumption that soil health improvement would lead to better water infiltration. Perhaps the short duration of no-till SSgH practice conducted here is too short to modify the soil infiltration rate.

Another interest in improving soil health is to examine if it can lead to the suppression of PPNs. While no-till practice is not allowing SSgH biofumigation to perform well, the CCA also showed no top-down regulation of the omnivorous nematodes against the PPNs. Rather, bottom-up support from the total abundance of PPNs (Herb) was observed to increase the abundance of omnivorous nematodes at 3 weeks after SSgH termination. Unfortunately, no predatory nematode was present in this field, which might explain the lack of top-down regulation of the PPNs since omnivorous nematodes were demonstrated to be less efficient for root-knot and reniform nematode predation [85].

Despite the lack of top-down regulation, it is encouraging that soil edaphic factors and soil health indicators (EI, SI, TPLFA, and CO_2_) were still negatively related to the abundance of root-knot, reniform, and total PPNs in the soil at 2 weeks after SSgH termination and to root-gall index (RGI) on eggplant at 3 months after SSgH termination. These could be explained by a shift in the microbial profile over time. Initially, a positive relation between the abundance of PPNs (reniform or RKNs) with microbial profile indices (GP/GN, S/U, and ACT) at 2 weeks after SSgH termination. This means the abundance of PPNs in the soil was associated with stressful soil conditions dominated by Gram-positive bacteria or actinomycetes [71]. Gram-positive bacteria such as *Bacillus* spp. and *Pasteuria penetrans* constitute important biological control agents against PPNs [86]. However, usually the population densities of these bacteria occurring naturally in the field are nematode-host-densities-dependent [87], which is why the relationship between PPN abundance and GP or GP/GN was initially positive. However, toward the third month after SSgH termination, the abundance of PPNs was negatively related to GP/PN, which might suggest a suppression of PPNs by GP.

The total yield of eggplant was unexpectedly not affected by any SSgH treatments or soil physical properties or negatively related to nematode diversity, fungivorous nematode abundance, SSgH biomass at cover crop termination, VSM, etc. Only the abundance of omnivorous and reniform nematodes was positively related to crop yield. This is the short-fall of practicing no-till farming for soil health management, as it often takes time to improve crop yield through no-till farming, similar to the meta-analysis results obtained by Pittelkow et al. [88].

## 5. Conclusions

The current studies clearly demonstrated that the biofumigation potential of SSgH cover crop could depend on various factors including the varieties, age of cover crop when terminated, and whether the SSgH residues were soil-incorporated. Among the varieties tested, energy sorghum ‘NX2’ (=‘NX-D-61’) and the hybrid ‘LA’ (=‘Latte’) were found to be highly suppressive to the development of root-knot nematodes into females even at 3 months old if the residues were soil-incorporated in the greenhouse trials. While terminating SSgH cover crops improved soil health parameters (edaphic factors, microbial profiling, and nematode community indices) within one cropping cycle, it did not lead to suppression of root-knot and reniform nematode population densities in the soil nor lead to better eggplant yield. However, there is a clear trend that (1) an increase in abundance of omnivorous nematodes would lead to higher eggplant yield; (2) an improvement in soil health could lead to a reduction in root-gall formation in eggplant; and (3) an increase in GP/GN would result in a lower abundance of PPNs in the soil. Future SSgH cover crop research should examine soil incorporation of SSgH residues in a minimum tillage system rather than no-till to allow for biofumigation to be performed. A longer-term study is needed to allow changes in microbial profiles toward achieving a nematode antagonistic effect. Nonetheless, microbial profiling using PLFA provided an indicative analysis of changes in the microbial biomass to SSgH no-till treatment, even within one cropping cycle. NX2 stood out to have improved the soil microbial profile toward a less stressful environment quicker than other varieties tested, but other SSgH varieties eventually also reduce stress in the microbial community, except for NX1. Bottom-up regulation by the combination of all soil microbial profiles might be shaping the nematode community and resulting in an improved soil edaphic factor in the shorter term, but a top-down control of PPNs might not be realized until a long-term soil health management system is installed.

## Figures and Tables

**Table 1:** SSgH varieties and their characteristic features.

Cultivar	Type	Characteristics
Big Kahuna Plus BMR (BKP)	Forage sorghum	Brown mid-rib trait with less lignin for increased digestibility in cattle, photoperiod sensitive, 2.4–2.7 m tall, late maturing.
Cow Vittles II (CV)	Forage sorghum	High yield potential, early seedling vigor.
Bundle King (BK)	Forage sorghum	Male sterile (no grain head), exceptional sweetness, large stems (less lodging), 2.4–2.7 m tall.
Latte (LA)	Sorghum–sudan hybrid	Late maturing, drought resistance, 2.1–2.4 m tall.
512 × 14 (512)	Sorghum–sudan hybrid	Conventional, excellent anti-lodging ability, hay grazing silage, long season.
NX 4264 (NX1)	Energy sorghum	Large biomass, long season hybrid, 4.5–6.1 m tall, dry stalk at the time of harvest.
NX-D-61 (NX2)	Energy sorghum	Large biomass, photoperiod neutral, 3–3.9 m tall, medium early maturing.

**Table 2:** Effect of SSgH age on the development of root-knot nematode female in roots of mustard green in the greenhouse trials.

Age	Trial I	Trial II
1 month old	3.26 ± 0.93 a ^z^	22.28 ± 6.01a
2 month old	19.40 ± 3.14 b	24.53 ± 3.96 b
3 month old	24.78 ± 2.24 c	37.22 ± 6.95 c

^z^ Means ± standard error (*n =* 56). Values with the same letters in a column are not significantly different at *p* *≤* 0.05, according to Waller–Duncan *k*-ratio (*k* = 100) *t*-test.

**Table 3:** Effect of SSgH cover crop on the abundance of nematode trophic groups and nematode community indices throughout the SSgH–eggplant cropping cycle.

Parameters				Treatments				
	BG	512	LA	BK	BKP	CV	NX1	NX2
*Abundance*	-----------250 cm^3^-----------							
Root-knot	447 ± 153 ab ^y^	966 ± 374 bc	1556 ± 570 a	957 ± 312 a	966 ± 361 ab	923 ± 330 c	812 ± 276 a	725 ± 232 ab
Reniform	800 ± 193 a	666 ± 86 a	595 ± 90 a	376 ± 43 a	568 ± 115 a	709 ± 118 a	466 ± 64 a	489 ± 82 a
Bacterivores	164 ± 30 b	293 ± 57 a^2^	206 ± 45 ab	179 ± 34 b	224 ± 50 b	325 ± 98 ab	220 ± 46 ab	310 ± 66 ab
Fungivores	69 ± 8 a	134 ± 22 a	82 ± 12 a	99 ± 17 a	90 ± 11 a	105 ± 15 a	117 ± 22 a	148 ± 28 a
Herbivores	1249 ± 234 a	1636 ± 415 a	2161 ± 594 a	1335 ± 330 a	1534 ± 389 a	1642 ± 391 a	466 ± 64 a	489 ± 82 a
Omnivores	4 ± 1 b	16 ± 6 a	15 ± 5 ab	16 ± 5 a	18 ± 6 a	8 ± 2 ab	11 ± 4 ab	19 ± 6 a
*Indices*								
Richness	8 ± 0 c	10 ± 1 a	9 ± 0 ab	10 ± 1 ab	9 ± 1 bc	9 ± 1 bc	10 ± 1 ab	10 ± 1 a
Diversity	2.57 ± 0.26 a	2.84 ± 0.27 a	2.48 ± 0.25 a	2.77 ± 0.27 a	2.78 ± 0.20 a	2.41 ± 0.20 a	3.03 ± 0.29 a	2.99 ± 0.26 a
EI (%) ^z^	1.96 ± 0.03 a	2.02 ± 0.05 a	2 ± 0.04 a	2.02 ± 0.05 a	2 ± 0.04 a	1.97 ± 0.05 a	1.96 ± 0.04 a	1.99 ± 0.06 a
SI (%)	45.58 ± 2.72 a	47.19 ± 3.47 a	48.52 ± 3.04 a	50.75 ± 3.39 a	50.51 ± 3.97 a	53.02 ± 3.74 a	54.81 ± 3.02 a	52.18 ± 4.05 a
MI (%)	0.36 ± 0.04 a	0.33 ± 0.03 a	0.37 ± 0.04 a	0.38 ± 0.04 a	0.39 ± 0.05 a	0.4 ± 0.04 a	0.39 ± 0.04 a	0.37 ± 0.04 a
CI (%)	11.39 ± 3.87 b	23.97 ± 4.29 ab	20.52 ± 4.13 ab	24.34 ± 4.33 a	24.49 ± 4.27 a	22.33 ± 4.34 ab	20.31 ± 4.79 ab	25.42 ± 4.43 a

^y^ Means ± standard error (*n =* 28) are averaged from repeated measures over seven sampling dates. Values followed by the same letter(s) in a row are not different based on Waller–Duncan *k*-ratio (*k* = 100) *t*-test. ^z^ EI = Enrichment index; SI = Structure index; MI = Maturity index; CI = Channel index.

**Table 4:** Effect of SSgH on soil microbial profile based on PLFA.

Parameters				Treatments				
	BG	512	LA	BK	BKP	CV	NX1	NX2
---27 August 2020---								
*Abundance*								
TPLFA(nmole/g) ^z^	40.7 ± 4.8 c ^y^	55.6 ± 4.6 b	78.1 ± 3.5 a	55.1 ± 6.2 b	60.4 ± 7.6 b	60.1 ± 3.6 b	53.4 ± 2.5 bc	65.4 ± 7.5 ab
GN (%)	34.6 ± 0.4 b	36.3 ± 0.8 ab	36.7 ± 0.5 ab	36.8 ± 1 ab	35.2 ± 1.5 b	34.7 ± 0.7 b	38 ± 0.9 a	36.1 ± 1.2 ab
GP (%)	38.7 ± 0.7 a	36.9 ± 1.2 a	36.8 ± 0.2 a	39.4 ± 2.9 a	38 ± 0.8 a	37.1 ± 0.4 a	38.5 ± 2.9 a	35.5 ± 0.7 a
AMF (%)	3.9 ± 0.1 a	3.9 ± 0.1 a	4.2 ± 0.2 a	4 ± 0.2 a	4.1 ± 0.3 a	3.9 ± 0.2 a	4.2 ± 0.1 a	4 ± 0.1 a
non-AMF (%)	2.9 ± 0.7 b	3.9 ± 0.8 ab	4 ± 0.2 ab	3.7 ± 0.4 ab	4.1 ± 0.8 ab	5.4 ± 0.9 a	3.7 ± 0.5 ab	5.4 ± 1.2 a
EUK (%)	0.9 ± 0.3 b	1.4 ± 0.3 ab	2.3 ± 0.4 ab	0.9 ± 0.1 b	1.6 ± 0.3 ab	1.7 ± 0.2 ab	1.4 ± 0.3 ab	2.5 ± 0.6 a
*Ratios*								
GP/GN	1.5 ± 0 a	1.3 ± 0 b	1.2 ± 0 b	1.3 ± 0 b	1.3 ± 0 b	1.3 ± 0 b	1.2 ± 0 b	1.2 ± 0 b
F/B	0.09 ± 0.01 b	0.11 ± 0.01 ab	0.11 ± 0 ab	0.11 ± 0.11 ab	0.12 ± 0.01 ab	0.13 ± 0.01 a	0.11 ±0.01 ab	0.13 ± 0.02 a
S/U	1.7 ± 0.1 a	1.4 ± 0.1 ab	1.3 ± 0 b	1.5 ± 0 ab	1.4 ± 0 b	1.4 ± 0 ab	1.4 ± 0 ab	1.4 ± 0.1 b
M/P	12.1 ± 3 a	7.2 ± 1.2 ab	5.8 ± 0.5 b	7.6 ± 1.1 ab	6.7 ± 1.1 ab	5.1 ± 0.7 b	7.3 ± 1.1 ab	4.9 ± 0.7 b
PD/PR	0.01 ± 0.01 a	0.02 ± 0.01 a	0.04 ± 0 a	0.02 ± 0 a	0.03 ± 0 a	0.03 ± 0 a	0.02 ± 0 a	0.04 ± 0.01 a
---10 December 2020---								
*Abundance*								
TPLFA(nmole/g)	41.5 ± 2.9 b	51.7± 10.7 ab	77.6 ± 18.2 a	52.6 ± 6.1 abc	54.2 ± 12.3 abc	60.1± 11.2 ab	34.8 ± 3.5 c	57 ± 10.7 abc
GN (%)	33.6 ± 0.4 a	33.2 ± 1.2 a	33.7 ± 1.5 a	33.3 ± 0.7 a	33.7 ± 1.1 a	33.9 ± 0.7 a	32.7 ± 0.1 a	35.6 ± 0.4 a
GP (%)	47.4 ± 0.5 a	39 ± 1.5 d	35 ± 1.8 e	39.7 ± 2 cd	39.4 ± 2.9 d	37.4 ± 0.9 de	45.8 ± 0.7 ab	43.2 ± 1.4 bc
AMF (%)	4.9 ± 0.2 a	2.8 ± 0.5 bcd	3.6 ± 0.4 ab	1.6 ± 0.2 d	3.1 ± 0.6 bc	3.3 ± 0.5 bc	3.8 ± 0.7 ab	2.1 ± 0.7 cd
non-AMF (%)	9.7 ± 1.3 a	4.5 ± 0.8 a	6.1 ± 0.9 a	3.5 ± 1.1 a	5.2 ± 1.3 a	4.6 ± 0.8 a	17.2 ± 10.4 a	3.5 ± 2.1 b
EUK (%)	1.8 ± 0.3 c	4 ± 0.6 abc	8 ± 3.3 a	6.1 ± 1.3 ab	5.5 ± 1.3 ab	4.4 ± 0.4 abc	1.1 ± 0.4 d	3.3 ± 0.7 bc
*Ratios*								
GP/GN	1.9 ± 0 a	1.5 ± 0.2 bc	1.3 ± 0 c	1.6 ± 0.1 b	1.6 ± 0.2 b	1.5 ± 0.1 bc	2 ± 0a	1.6 ± 0.1 b
F/B	0.2 ± 0 a	0.1 ± 0 abc	0.1 ± 0 ab	0.1 ± 0 bc	0.1 ± 0 abc	0.1 ± 0 abc	0.4 ± 0.3 a	0.1 ± 0 c
S/U	7.8 ± 0.8 ab	2.4 ± 0.6 de	1.5 ± 0.2 e	2.9 ± 0.6 cd	4.9 ± 2.8 cd	2 ± 0.3 de	9 ± 0.7 a	5.4 ± 1.9 bc
M/P	6.6 ± 0.8 b	3.1 ± 0.2 bc	2.5 ± 0.5 c	2.5 ± 0.6 c	2.7 ± 0.4 c	3.1 ± 0.2 bc	10.6 ± 2.4 a	4.6 ± 1.2 bc
PD/PR	0.02 ± 0 b	0.1 ± 0 ab	0.1 ± 0.07 a	0.1 ± 0 a	0.1 ± 0 a	0.1 ± 0 ab	0.02 ± 0 c	0.1 ± 0 ab

^y^ Means (*n =* 4) followed by the same letter(s) in a row are not different based on Waller–Duncan *k*-ratio (*k* = 100) *t*-test. ^z^ TPLFA = Total phospholipid fatty acid representing total microbial biomass in nanomoles per gram of soil; Microbial groups such as GN = Gram-negative bacteria; GP = Gram-positive bacteria; AMF = Arbuscular mycorrhizal fungi; non-AMF = non-arbuscular mycorrhizal fungi; EUK = Eukaryotes are percentage of signature fatty acid peaks; GP/GN = ratio of Gram + ve bacteria to Gram − ve bacteria; F/B = ratio of fungi to bacteria; S/U = ratio of saturated to unsaturated fatty acids; M/P = ratio of monounsaturated to polyunsaturated fatty acids; PD/PR = ratio of predator to prey (Protozoa/bacteria).

**Table 5:** Effect of SSgH cover cropping on eggplant growth and total yield.

	Treatments							
Parameters	BG	512	LA	BK	BKP	CV	NX1	NX2
Plant height (cm)	11.7 ± 1.91 a ^y^	16 ± 1.23 a	14.02 ± 1.5 a	13.9 ± 1.75 a	13.71 ± 0.86 a	14.34 ± 1.74 a	14.5 ± 2.01 a	14.45 ± 0.83 a
Total fruit wt (kg)	2.47 ± 0.33 a	3.64 ± 0.44 a	2.15 ± 0.23 a	3.4 ± 0.76 a	2.45 ± 0.52 a	3.63 ± 0.43 a	2.6 ± 0.61 a	3.63 ± 0.53 a
Total fruit no.	27 ± 3.72 a	31 ± 1.89 a	25 ± 2.1 a	28 ± 6.28 a	30 ± 4.7 a	38 ± 5.68 a	27 ± 4.56 a	29 ± 2.32 a
Root wt (kg)	0.07 ± 0.01 b	0.1 ± 0.01 ab	0.09 ± 0.01 ab	0.11 ± 0.01 a	0.08 ± 0.01 ab	0.10 ± 0.01 ab	0.11 ± 0.01 ab	0.1 ± 0.01 ab
RGI (0–10) ^z^	5.43 ± 0.43 a	5.50 ± 0.23 a	6.13 ± 0.55 a	6.73 ± 0.22 a	5.33 ± 0.35 a	5.50 ± 0.51 a	5.98 ± 0.38 a	6.10 ± 0.41 a

^y^ Means ± standard error (*n =* 4) are from repeated measures over 10 sampling dates for total fruit number/plot and total fruit weight/plot. Means ± standard error (*n* = 16) for root-gall index, root weight, and plant height per plant/plot. Values followed by the same letter(s) in a row are not different based on Waller–Duncan *k*-ratio (*k* = 100) *t*-test. ^z^ RGI = Root-gall index on a 0–10 scale.

## Data Availability

Data are contained within the article and supplementary material available at figshare: https://doi.org/10.6084/m9.figshare.15070266 (accessed on 29 July 2021).

## Supplementary Materials

The following are available online at https://www.mdpi.com/article/10.3390/microorganisms9091831/s1, Table S1: Initial population of *Meloidogyne* spp. and *Rotylenchulus reniformis* per 250 cm^3^ soil.
