# Supplementary material for: Exploiting the Innate Potential of Sorghum/Sorghum–Sudangrass Cover Crops to Improve Soil Microbial Profile That Can Lead to Suppression of Plant-Parasitic Nematodes
Source: Microorganisms. 2021 Aug 29;9(9):1831. doi: 10.3390/microorganisms9091831 (PMC8470355; doi:10.3390/microorganisms9091831)
Supplement: Supplementary file 1 [file microorganisms-09-01831-s001.zip › microorganisms-1339094-supplementary.pdf]

Table S1. Initial population of *Meloidogyne* spp. and *Rotylenchulus reniformis* per 250 cm^3 soil.

	Treatments							
Abundance	BG	512	LA	BK	BKP	CV	NX1	NX2
				----------- 250 cm^3 -----------				
Root-knot	553 ± 223a[y]	463 ± 148a	258 ± 49a	548± 232a	818 ± 383a	530 ± 217a	390 ± 143a	533± 160a
Reniform	15 ± 3a	10± 4a	13 ± 3a	20 ± 7a	30 ± 12a	15 ± 3a	13 ± 6a	20 ± 14a

[y]Means ± standard error (*n* = *4*) followed by the same letter(s) in a row are not different based on Waller-Duncan *k*-ratio (*k* = 100) *t*-test.
